# Effectiveness of Digital Medicines to Improve Clinical Outcomes in Patients with Uncontrolled Hypertension and Type 2 Diabetes: Prospective, Open-Label, Cluster-Randomized Pilot Clinical Trial

**DOI:** 10.2196/jmir.7833

**Published:** 2017-07-11

**Authors:** Juan Frias, Naunihal Virdi, Praveen Raja, Yoona Kim, George Savage, Lars Osterberg

**Affiliations:** ^1^ National Research Institute Los Angeles, CA United States; ^2^ Proteus Digital Health Redwood City, CA United States; ^3^ Stanford University, School of Medicine Stanford, CA United States

**Keywords:** digital medicine, hypertension, type 2 diabetes, patient engagement, medication adherence, therapeutic inertia

## Abstract

**Background:**

Hypertension and type 2 diabetes mellitus are major modifiable risk factors for cardiac, cerebrovascular, and kidney diseases. Reasons for poor disease control include nonadherence, lack of patient engagement, and therapeutic inertia.

**Objective:**

The aim of this study was to assess the impact on clinic-measured blood pressure (BP) and glycated hemoglobin (HbA1c) using a digital medicine offering (DMO) that measures medication ingestion adherence, physical activity, and rest using digital medicines (medication taken with ingestible sensor), wearable sensor patches, and a mobile device app.

**Methods:**

Participants with elevated systolic BP (SBP ≥140 mm Hg) and HbA1c (≥7%) failing antihypertensive (≥2 medications) and oral diabetes therapy were enrolled in this three-arm, 12-week, cluster-randomized study. Participants used DMO (includes digital medicines, the wearable sensor patch, and the mobile device app) for 4 or 12 weeks or received usual care based on site randomization. Providers in the DMO arms could review the DMO data via a Web portal. In all three arms, providers were instructed to make medical decisions (medication titration, adherence counseling, education, and lifestyle coaching) on all available clinical information at each visit. Primary outcome was change in SBP at week 4. Other outcomes included change in SBP and HbA1c at week 12, and low-density lipoprotein cholesterol (LDL-C) and diastolic blood pressure (DBP) at weeks 4 and 12, as well as proportion of patients at BP goal (<140/90 mm Hg) at weeks 4 and 12, medical decisions, and medication adherence patterns.

**Results:**

Final analysis included 109 participants (12 sites; age: mean 58.7, SD years; female: 49.5%, 54/109; Hispanic: 45.9%, 50/109; income ≤ US $20,000: 56.9%, 62/109; and ≤ high school education: 52.3%, 57/109). The DMO groups had 80 participants (7 sites) and usual care had 29 participants (5 sites). At week 4, DMO resulted in a statistically greater SBP reduction than usual care (mean –21.8, SE 1.5 mm Hg vs mean –12.7, SE 2.8 mmHg; mean difference –9.1, 95% CI –14.0 to –3.3 mm Hg) and maintained a greater reduction at week 12. The DMO groups had greater reductions in HbA1c, DBP, and LDL-C, and a greater proportion of participants at BP goal at weeks 4 and 12 compared with usual care. The DMO groups also received more therapeutic interventions than usual care. Medication adherence was ≥80% while using the DMO. The most common adverse event was a self-limited rash at the wearable sensor site (12%, 10/82).

**Conclusions:**

For patients failing hypertension and diabetes oral therapy, this DMO, which provides dose-by-dose feedback on medication ingestion adherence, can help lower BP, HbA1c, and LDL-C, and promote patient engagement and provider decision making.

**Trial Registration:**

Clinicaltrials.gov NCT02827630; https://clinicaltrials.gov/show/NCT02827630 (Archived by WebCite at http://www.webcitation.org/6rL8dW2VF)

## Introduction

Hypertension (HTN) and diabetes mellitus are major risk factors for cardiac diseases, stroke, and kidney diseases [1-5]. Despite the widespread availability of effective treatments, approximately half of treated patients do not have adequate blood pressure (BP) or glycemic control [4,6,7]. Poor medication adherence, lack of patient engagement, and therapeutic inertia are major contributors to patients not reaching their therapeutic targets [8-12]. Medication nonadherence alone costs US $290 billion annually in the United States and is difficult to assess and improve [13,14].

The psychology literature suggests that human beings in general are poor intuitive statisticians in that they cannot estimate their risk for consequences related to nonadherence and poor disease control [15]. Proteus Digital Health (Redwood City, CA, USA) hypothesized that this problem might be addressed by a common solution: detailed feedback to patients and physicians of actual dosing behavior. This would present patients with a clear adherence target while allowing physicians to discern lack-of-response calling for dosage or medication changes from patient nonadherence.

**Figure 1:**
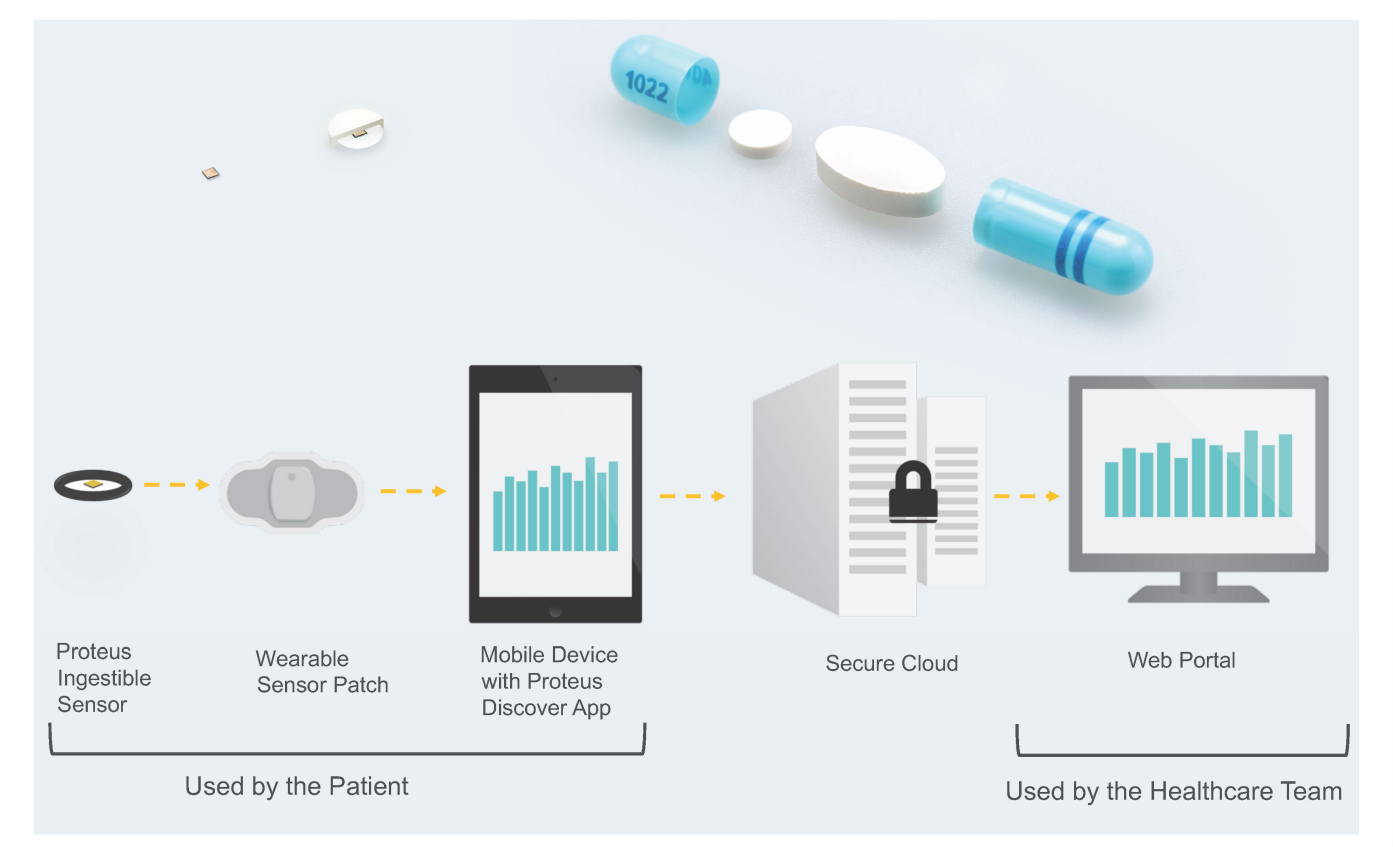
Top left: ingestible sensor and ingestible sensor pill. Top right: coencapsulation of a medication with an ingestible sensor pill. Bottom: components of the DMO and data flow.

Proteus Discover, a digital medicine offering (DMO) from Proteus Digital Health, was designed specifically to provide feedback for medication taking and other health behaviors to both patients and providers. It consists of an ingestible sensor (contained inside a placebo pill), an adhesive wearable sensor patch, a patient mobile app, and a provider Web portal. After being swallowed, the ingestible sensor is activated and sends a signal with a specific code that is detected by the patch. When the ingestible sensor pill is taken with medication (now a digital medicine), the DMO can measure medication ingestion adherence. To ensure that the ingestible sensor and medication are taken simultaneously, the two can be co-encapsulated by a pharmacist (as was done during this study). The patch also measures activity, body angle, heart rate, and step count. Data from the patch are transmitted to a mobile device (eg, mobile phone) and then to the cloud. Patients can visualize the DMO data on their mobile device via an app and providers can view summaries of the DMO data for their patients on the Web portal. The mobile device app also prompts the patient to take their medication doses as scheduled. The goal of the DMO is to improve clinical outcomes through better patient self-care, enhanced patient-provider dialog, and data-driven optimization of therapy. (See Figure 1 for an overview of the DMO.)

Prior clinical studies demonstrated the accuracy, safety, and feasibility of using the DMO in patients across a range of medical conditions, including HTN and type 2 diabetes mellitus (T2DM), and suggested this DMO can identify reasons for uncontrolled HTN and help patients achieve BP control [16-21]. However, these uncontrolled studies did not focus on disease control. A prior hypertension registry study conducted in several primary care centers in the United Kingdom demonstrated the ability of a prior version of the DMO with no patient feedback (ie, digital medicines plus patch) to uncover a root cause for uncontrolled HTN in all participants after 2 weeks of use. Additionally, 37% of participants achieved BP control after 2 weeks with no adjustments to their antihypertensive medications [20].

In this study, patients with uncontrolled T2DM and HTN with current therapy were offered the DMO or usual care (to compare with the current standard that patients receive today). The design of the HTN registry was used to determine the duration of DMO use in this study; the first 2 weeks of DMO use allowed providers to understand the root cause for elevated BP (nonadherence, inadequate medication, or both) and the subsequent 2 weeks allowed the provider to see the effect of the medical decision on BP after the first 2 weeks [20]. The primary objective was to study the effect of the DMO on BP. Additional objectives were to assess the effect on glycemic and lipid control, engagement, and provider decision making.

## Methods

### Study Design and Participants

This 12-week, open-label, prospective, cluster-randomized, controlled, three-arm pilot study was executed at 13 outpatient primary care sites across California and Colorado. There were three additional sites that did not enroll any patients. Sites were selected based on the size of their HTN and T2DM population. Use of a cluster-randomized design mitigated the risk of investigator bias by ensuring providers cared for participants assigned to only one arm. Sites were randomized to the treatment arms: DMO use for 4 weeks (4-week DMO), DMO use for 12 weeks (12-week DMO), or usual care. Enrolled participants were assigned to the treatment arm of their clinical site.

Adults with uncontrolled HTN (systolic BP [SBP] ≥140 mm Hg) and T2DM (glycated hemoglobin A_1c_ [HbA_1c_] ≥7%) who failed treatment with two or more antihypertensive medications available as part of the DMO medicine panel (Table 1) or dose-equivalent medicines from the same classes, and metformin and/or a sulfonylurea were eligible for enrollment. Participants either needed to be able to use a mobile phone or tablet or the investigator determined the participant could learn to use a smart mobile device. Investigators also assessed whether their participants could be treated for HTN during the study period using the DMO medication panel exclusively; if medically necessary, participants could be prescribed off-panel antihypertensive medicines. Exclusion criteria included body mass index (BMI) >40 kg/m^2^, skin sensitivity to adhesive medical tape or metals, active or chronic dermatitis, secondary causes for uncontrolled HTN or T2DM, evidence of hypertensive emergency, and use of insulin or other injectables to treat T2DM within the past year.

Copernicus Group Independent Review Board, a central institutional review board, approved and monitored the study. Participants provided written informed consent prior to screening and were compensated (US $150 to US $525 based on study arm and site-specific guidelines for participant compensation) for participation.

During the study, changes were made to make the inclusion and exclusion criteria less restrictive to promote recruitment. Notably, we included a Spanish version of the consent form and removed an exclusion of non-English speakers.

### Interventions

Investigators were instructed to make medication changes and to provide patient education and counseling as clinically appropriate (versus using specific dose-escalation protocols) to ensure the decisions were similar to those in a real clinical practice setting. Blood pressure recorded at each visit was the mean of two or more BP measurements obtained using the recommended measurement guidelines from the American Heart Association [22]. Participants had their BP measured after 5 or more minutes of rest, comfortably seated in a quiet room with their feet touching the floor. Each BP measurement was taken at least 1 minute apart. If the first two BP readings were more than 5 mm Hg different, then the BP was measured at least two more times; the mean of all BP values from one visit were used as the final reading. Laboratory test results were drawn at screening, and at weeks 4 and 12, for HbA_1c_ (screening and week 12), fasting plasma glucose (FPG), total cholesterol, and directly measured low-density lipoprotein cholesterol (LDL-C). Participants also completed the 10-question Patient Activation Measure (PAM), a validated measure of patient activation that includes person’s beliefs, motivation, and actions for self-care at these visits [9,23,24]. During each visit, participants and investigators jointly reviewed the data and collaboratively set goals for medication adherence, physical activity, and rest.

Participants in the DMO arms were prescribed DMO for either 4 or 12 weeks and medicines co-encapsulated with ingestible sensors (see digital medicine panel in Table 1). Participants were allowed to switch to medications on the digital medicine panel in a dose-equivalent manner from the same drug classes. The DMO investigators were instructed to review DMO reports on the Web portal during study visits.

**Table 1:** Digital medicine offering panel.^a^

Therapeutic area and class		Medication	Doses
**Hypertension**			
	Angiotensin-converting enzyme inhibitor	Lisinopril	10 mg, 20 mg, 40 mg
	Angiotensin receptor blocker	Losartan	100 mg
	Thiazide diuretic	HCTZ	12.5 mg, 25 mg
	Dihydropyridine calcium channel blocker	Amlodipine	5 mg
**Hypercholesterolemia**			
	Statin	Atorvastatin	20 mg
**Diabetes**			
	Biguanide	Metformin	500 mg
	Sulfonylurea	Glipizide	5 mg

^a^Participants could take more than one medication dose at any one time (eg, ingesting two atorvastatin 20 mg capsules to get a total dose of 40 mg).

All investigators could titrate medications, provide patient education, and/or counseling at any time during the study as per usual care based on all available clinical data, with the exception that investigators in the DMO arms were instructed to await the DMO report from the first 2 weeks of DMO use before making changes to the antihypertensive medications (or other medical decision) to try to ensure this decision was made using the DMO data.

Providers trained participants on use of the DMO and, along with customer support provided by the sponsor, assisted participants in troubleshooting issues with the DMO.

### Study Outcomes

The primary endpoint was change in SBP from baseline to week 4. Secondary endpoints included changes from baseline in SBP and HbA_1c_ at week 12, changes in diastolic BP (DBP) and FPG at weeks 4 and 12, proportion of participants at BP goal (SBP <140 mm Hg and DBP <90 mm Hg) at weeks 4 and 12, medication adherence rate, and mean daily step count and duration of physical activity and rest (DMO only), and medical decisions. Exploratory outcomes included change in LDL-C from baseline at weeks 4 and 12 in patients using digital atorvastatin (DMO arms) or any statin (usual care) and change in PAM score.

### Statistical Analysis

Target enrollment in this pilot study was 120 participants to ensure at least 90 evaluable participants at the end of the study. This study was primarily performed to understand the effect size of the DMO intervention. Although there was prior data from the hypertension registry study, the product and study design were different: participants in the hypertension registry study used a DMO without feedback for 2 weeks. It was hypothesized that with feedback to the participant and provider and a longer intervention, the effect size would be larger. Due to the pilot nature of the study, there were no a priori hypotheses for this study; *P* values are not reported on study outcome data. *P* values were only calculated for baseline differences between groups.

Values and change for continuous variables were summarized descriptively (mean and SE) and 95% confidence intervals (95% CI) were calculated for all changes. Differences between groups were calculated using a mixed-effects regression model that incorporated covariates found to be significant in the model: baseline value, gender, age (<65 years vs ≥65 years), and race (African American vs other). Proportions as well as differences between groups were summarized descriptively.

The SE was adjusted for cluster (study site) effects as well as the intracluster correlation coefficient (ICC) calculated using a one-way analysis of variance to adjust for any imbalances between and within clusters. Confidence intervals were calculated for differences between groups.

Participants with at least one follow-up BP were included in this modified intention-to-treat (ITT) analysis, which represented the minimal data needed for a pre/post comparison for each participant. Missing data were handled using last observation carried forward. Safety assessments were performed on all enrolled participants. Analyses were performed for 4-week DMO, 12-week DMO, and combined DMO (both DMO arms combined). Because both DMO groups had the same intervention for the first 4 weeks, the two groups were combined for all week 4 endpoints and measures (eg, mean medication adherence).

**Figure 2:**
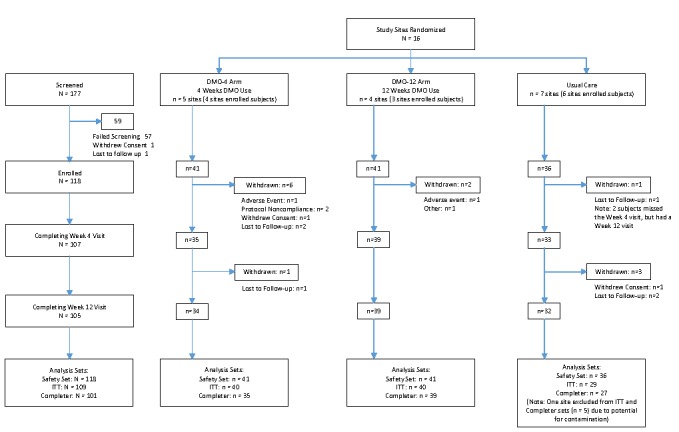
CONSORT flow diagram of participants.

**Table 2:** Demographics and baseline characteristics of participants (N=109).

Parameter	4-week DMO (n=40)	12-week DMO (n=40)	Combined DMO (n=80)	Usual care (n=29)
Age (years), mean (SE)	58.8 (1.4)	56.7 (1.8)	57.8 (1.1)	61.6 (1.7)
Female, n (%)	21 (53)	24 (60)	45 (56)	10 (35)
African American, n (%)	11 (28)	3 (8)	14 (18)	3 (10)
Caucasian, n (%)	29 (73)	24 (60)	53 (66)	19 (66)
Asian, n (%)	0 (0)	13 (33)	13 (16)	2 (7)
Hispanic ethnicity (includes all races), n (%)	22 (55)	15 (38)	37 (46)	14 (45)
Income ≤ US$20,000, n (%)	23 (58)	21 (53)	44 (55)	18 (62)
Education <high school, n (%)	18 (45)	6 (15)	24 (30)	10 (34)
Employed, n (%)	18 (45)	24 (60)	42 (53)	9 (31)
Weight (kg), mean (SE)	91.5 (5.9)	85.7 (3.4)	88.6 (3.3)	89.7 (4.7)
BMI (kg/m^2^), mean (SE)	32.8 (1.4)	30.7 (0.9)	31.8 (0.9)	31.3 (1.0)
Systolic BP (mm Hg), mean (SE)	152.2 (1.6)	146.5 (0.8)^a^	149.3 (1.5)^a^	155.4 (3.0)
Diastolic BP (mm Hg), mean (SE)	90.5 (2.8)	82.0 (5.1)	86.2 (3.2)	83.9 (2.9)
HbA_1c_ (%), mean (SE)	8.8 (0.3)	8.5 (0.2)	8.7 (0.2)	8.3 (0.4)
FPG (mg/dL), mean (SE)	174.2 (13.6)	191.4 (16.2)	182.8 (9.9)	165.0 (8.5)
LDL-C (mg/dL), mean (SE)	110.7 (5.3)	107.1 (6.6)	108.9 (3.9)	99.1 (6.2)
HDL-C (mg/dL), mean (SE)	47.8 (2.6)	45.2 (1.5)	46.5 (1.4)	40.6 (2.5)
Triglycerides (mg/dL), mean (SE)	211.2 (28.1)	195.7 (17.3)	203.4 (16.2)	226.1 (36.2)
Total cholesterol (mg/dL), mean (SE)	190.2 (6.5)	175.3 (6.0)	182.8 (4.5)	174.4 (13.2)

^a^Difference compared to usual care was statistically significant (*P*<.05).

**Figure 3:**
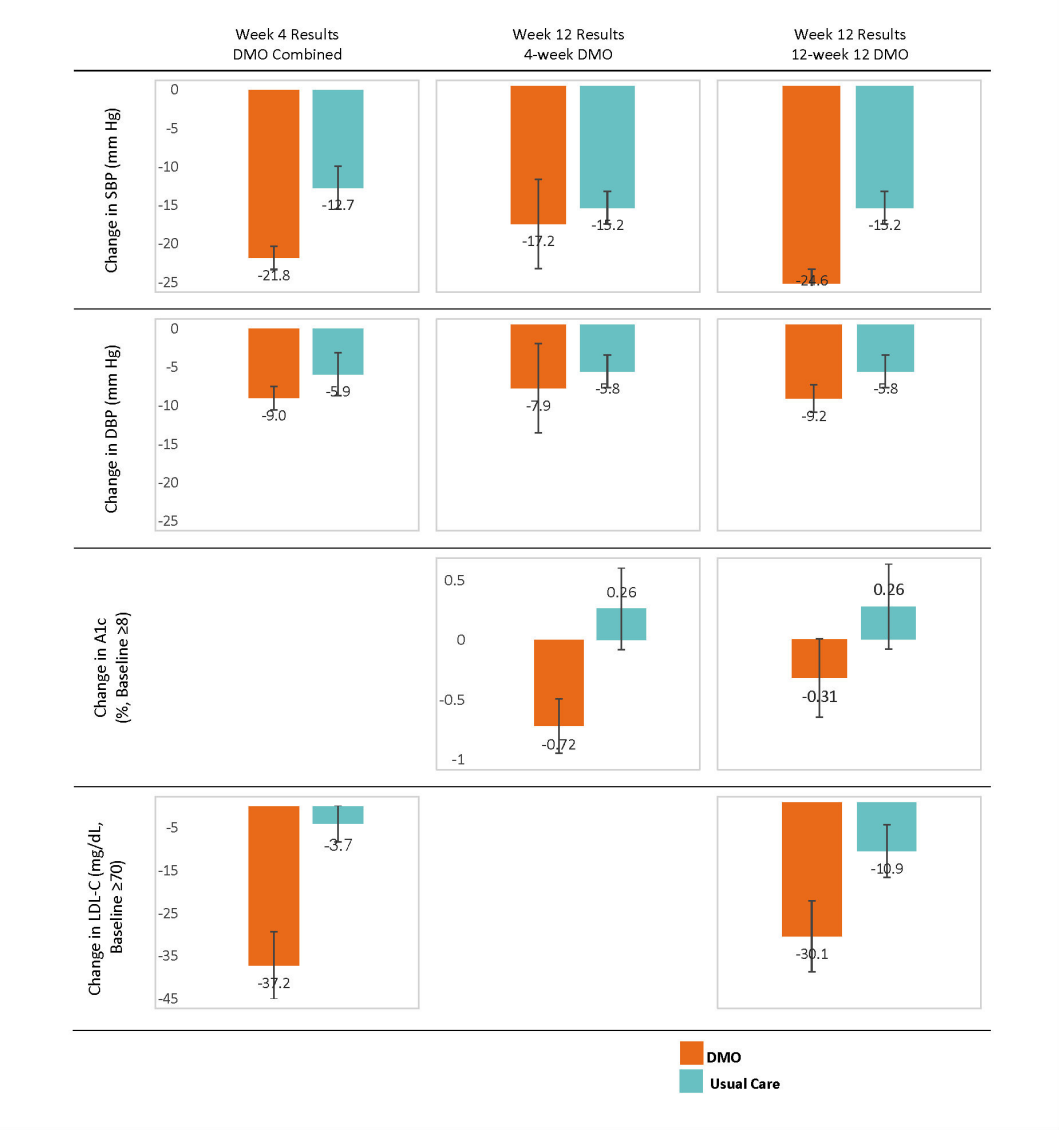
Highlighted clinical results for changes in systolic and diastolic blood pressure (SBP and DPB), glycated hemoglobin (HbA1c), and low-density lipoprotein cholesterol (LDL-C) for the combined digital medicine offering (DMO) groups at week 4, week-4 DMO, and 12-week DMO. Error bars represent SE.

Medication adherence data and medical decisions (provider treatment decisions in response to DMO data) were summarized descriptively. Medication adherence was calculated only for the DMO users because measurement of medication adherence was an intrinsic aspect of the intervention.

Analyses of efficacy variables were performed on R version 3.2.2 with lme4 version 1.1.11 for building generalized linear mixed models. An interim analysis was performed in October 2015 to get preliminary data on the primary outcome.

## Results

Between June and October 2015, 118 participants were enrolled across the 13 sites; 107 participants completed the week 4 visit and 105 completed the week 12 visit by December 30, 2015. Three usual care participants missed the week 4 visit, but were included in the analysis because they returned for the week 12 visit. One usual care site with five participants was not included in the final analysis over concern about violation of the cluster randomization. This usual care site was activated in September and was joined by the lead study coordinator from a 4-week DMO site previously activated in May; this study coordinator had intervened with both DMO and usual care participants. The final modified-ITT group included 109 participants (40 in 4-week DMO, 40 in 12-week DMO, and 29 in usual care) (Figure 2).

The study included a large portion of low-income participants (56.9%, 62/109 earned ≤ US $20,000 per year) and minorities (52.3%, 57/109 Hispanics) (Table 2). In addition, 22.0% (24/109; 25%, [20/80] in DMO and 14%, [4/29] in usual care) had psychiatric comorbidities.

### Primary Outcome

At week 4, combined DMO had a mean change in SBP from baseline of –21.8 (SE 1.5) mm Hg compared to –12.7 (SE 2.8) mm Hg for usual care (combined DMO–usual care: mean –9.1, SE 2.9, 95% CI –14.8 to –3.3 mm Hg; ICC=0; adjusted difference: mean –10.0, SE 3.1, 95% CI –16.1 to –3.9 mm Hg; effect size=0.69) (Figure 3). A sensitivity analysis showed that excluding the one usual care site did not impact the primary outcome; the change in SBP for usual care with the excluded site was mean –14.0 (SE 2.7; difference from combined DMO: –7.8, SE 2.8, 95% CI –13.3 to –2.3 mm Hg).

### Secondary Outcomes

#### Hypertension

At week 4, a greater proportion of DMO participants achieved their BP goal (81%, 65/80) compared with usual care (33.3%, 9/27; mean difference 47.9%, SE 15.0%, 95% CI 18.5%-77.3%) (Table 3). DMO participants also had a greater reduction in DBP compared with usual care, but the results were not significant. The 12-week DMO group continued to show larger reductions in SBP from baseline (mean –24.6, SE 1.7 mm Hg), which was statistically larger compared to usual care (mean –15.2, SE 2.0 mm Hg; mean difference –9.4, SE 2.7, 95% CI –14.6 to –4.2 mm Hg). At week 12, 98% (39/40) of 12-week DMO participants achieved their BP goal compared with 51.7% of usual care participants (mean difference 45.8%, 95% CI 7.1% to 84.5%). The 4-week DMO group also had greater reductions in SBP and DBP at week 12 than usual care, but the results were not statistically significantly different (Table 4).

#### Diabetes

At week 12, DMO had a nonsignificant difference in HbA_1c_ reduction compared to usual care (4-week DMO: mean –0.32%, SE 0.22%; 12-week DMO: mean –0.08%, SE 0.22%; usual care: mean 0.28%, SE 0.35%). For participants with a baseline HbA_1c_ of 8% of more (n=65; 4-week DMO: n=26, 12-week DMO: n=24, usual care group: n=15), both DMO groups showed larger HbA_1c_ decreases (4-week DMO: mean –0.72%, SE 0.23%; 12-week DMO: mean –0.31%, SE 0.31%) compared to an increase in the HbA_1c_ seen in the usual care group (mean 0.26%, SE 0.34%; difference from 4-week DMO 0.98%, 95% CI –1.72 to –0.24; difference from 12-week DMO –0.57%, 95% CI –1.53 to 0.39) (See Figure 3 and Table 3). Adjusted differences for the change in HbA_1c_ between each DMO group and usual care were almost 1%. There were no significant differences in change in FPG between the DMO and usual care groups.

**Table 3:** Summary of systolic and diastolic blood pressure (SBP and DBP), fasting plasma glucose (FPG), and glycated hemoglobin A_1c_ (HbA_1c_) results for usual care and combined digital medicine offering (DMO).

Outcome		Usual care	DMO (combined)		
		Value	Value	Difference,^a^ (95% CI)	Adjusted difference,^a^ (95% CI)
**SBP (mm Hg)**					
	Baseline, mean (SE)	155.4 (3.0)	149.3 (1.5)		
	Week 4, mean change (SE)	–12.7 (2.8)	–21.8 (1.5)	–9.1 (2.9); (–14.0, –3.3)	–10.0 (3.1); (–16.1, –3.9)
	Week 12, mean change (SE)	–15.2 (2.0)	–20.9 (3.4)	–4.6 (4.9); (–14.3, 5.1)	–4.8 (5.6); (–15.8, 6.3)
**DBP (mm Hg)**					
	Baseline, mean (SE)	83.9 (2.9)	86.2 (3.2)		
	Week 4, mean change (SE)	–5.9 (3.0)	–9.0 (1.6)	–3.4 (3.1); (–9.4, 2.7)	–2.4 (1.9); (–6.2, 1.3)
	Week 12, mean change (SE)	–5.8 (2.2)	–8.6 (2.2)	–2.4 (3.4); (–9.1, 4.4)	–1.2 (3.4); (–7.2, 4.8)
**Proportion at BP goal (%)**					
	Week 4, mean (SE)	33.3 (9.7)	81.2 (5.1)	47.9 (15.0); (18.5, 77.3)	N/A^b^
	Week 12, mean (SE)	51.7 (15.6)	80.0 (9.3)	28.3 (24.6); (–19.9, 76.5)	N/A^b^
**FPG (mg/dL)**					
	Baseline, mean (SE)	165.0 (13.6)	182.8 (9.9)		
	Week 4, mean change (SE)	13.4 (15.8)	–9.4 (14.3)	–22.7 (22.0); (–66.7, 21.4)	–14.4 (21.7); (–57.0, 28.3)
	Week 12, mean change (SE)	14.9 (12.0)	–4.9 (14.9)	–16.2 (22.1); (–59.5, 27.1)	–12.6 (20.1); (–52.0, 26.9)
**HbA_1c_ (%)**					
	Baseline, mean (SE)	8.28 (0.38)	8.66 (0.18)		
	Week 12, mean change (SE)	0.26 (0.35)	–0.19 (0.14)	–0.48 (0.29); (–1.04, 0.09)	–0.54 (0.41); (–1.3, 0.3)
**HbA_1c_ baseline ≥8% (%)^c^**					
	Baseline, mean (SE)	9.25 (0.31)	9.54 (0.19)		
	Week 12, mean change (SE)	0.26 (0.34)	–0.50 (0.20)	–0.77 (0.40); (–1.6, 0.02)	–0.94 (0.45); (–1.8, –0.1)

^a^Difference from usual care.

^b^N/A: Adjusted analysis was not performed.

^c^Usual care: n=15; DMO: n=50.

**Table 4:** Summary of systolic and diastolic blood pressure (SBP and DBP), fasting plasma glucose (FPG), and glycated hemoglobin A1c (HbA1c) results for 4-week and 12-week digital medicine offering (DMO) groups.

Outcome		4-week DMO			12-week DMO		
		Value	Difference,^a^ (95% CI)	Adjusted difference,^a^ (95% CI)	Value	Difference,^a^ (95% CI)	Adjusted difference,^a^ (95% CI)
**SBP (mm Hg)**							
	Baseline mean (SE)	152.2 (1.6)			146.4 (0.8)		
	Week 4 mean change (SE)	–21.5 (2.5)	–8.8 (3.5); (–15.7, –1.9)	–8.5 (3.8); (–15.8, –1.1)	–22.1 (1.8)	–9.4 (2.9); (–15.1, –3.6)	–11.3 (3.3); (–17.6, –4.9)
	Week 12 mean change (SE)	–17.2 (5.6)	–1.1 (5.9); (–12.6, 10.4)	–0.3 (6.2); (–12.5, 11.9)	–24.6 (1.7)	–9.4 (2.7); (–14.6, –4.2)	–11.0 (3.1); (–17.1, –4.9)
**DBP (mm Hg)**							
	Baseline mean (SE)	90.5 (2.8)			82.0 (5.1)		
	Week 4 mean change (SE)	–10.1 (1.6)	–4.3 (3.1); (–10.5, 1.8)	–1.6 (2.4); (–6.2, 3.0)	–7.8 (3.9)	–2.1 (5.2); (–12.2, 8.0)	–4.4 (2.5); (–9.4, 0.5)
	Week 12 mean change (SE)	–7.9 (3.3)	–1.8 (4.0); (–9.7, 6.1)	2.0 (4.0); (–4.6, 8.6)	–9.2 (3.6)	–3.1 (4.1); (–11.2, 4.9)	–5.9 (4.1); (–13.3, 1.5)
**Proportion at BP goal (%)**							
	Week 4 mean (SE)	72.5 (7.3)	39.2 (12.5); (14.7, 63.7)	N/A^b^	90.0 (6.7)	56.7 (16.4); (24.6, 88.7)	N/A^b^
	Week 12 mean (SE)	62.5 (9.3)	10.8 (23.4); (–35.1, 56.6)	N/A^b^	97.5 (2.5)	45.8 (19.8); (7.1, 84.5)	N/A^b^
**FPG (mg/dL)**							
	Baseline mean (SE)	174.2 (13.6)			191.4 (16.2)		
	Week 4 mean change (SE)	4.7 (9.6)	–10.3 (19.9); (–49.2, 28.6)	–9.8 (23.5); (–56.0, 36.3)	–22.8 (31.1)	–38.7 (32.0); (–101.4, 24.1)	–15.9 (30.7); (–76.1, 44.3)
	Week 12 mean change (SE)	20.9 (9.2)	6.8 (15.4); (–23.4, 37.0)	–0.5 (17.3); (–34.4, 33.4)	–28.9 (18.9)	–44.6 (21.8); (–87.4, –1.8)	–26.3 (22.9); (–71.2, 18.7)
**HbA**_1c_**(%)**							
	Baseline mean (SE)	8.79 (0.29)			8.53 (0.20)		
	Week 12 mean change (SE)	–0.32 (0.22)	–0.65 (0.44); (–1.52, 0.23)	–0.63 (0.54); (–1.69, 0.43)	–0.08 (0.22)	–0.35 (0.40); (–1.13, 0.42)	–0.50 (0.67); (–1.81, 0.81)
**HbA**_1c_**baseline ≥8% (%)^c^**							
	Baseline mean (SE)	9.78 (0.30)			9.29 (0.23)		
	Week 12 mean change (SE)	–0.72 (0.23)	–0.98 (0.38); (–1.72, –0.24)	–0.98 (0.45); (–1.86, –0.10)	–0.31 (0.31)	–0.57 (0.49); (–1.53, 0.39)	–0.98 (0.58); (–2.12, 0.16)

^a^Difference from usual care.

^b^N/A: Adjusted analysis was not performed.

^c^4-week DMO: n=26; 12-week DMO: n=24.

**Figure 4:**
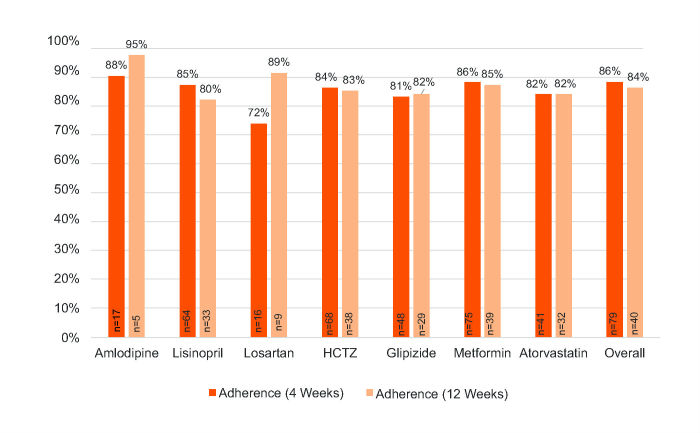
Ingestion adherence for DMO subjects measured by DMO. Note adherence for the first 4 weeks includes both 4-week DMO and 12-week DMO; adherence for 12 weeks includes only 12-week DMO.

#### Medical Decisions

The DMO providers made approximately 3 times more medical decisions per participant (mean 6.5, SD 5.3 DMO vs mean 2.7, SD 3.3 usual care). The DMO participants received more counseling, patient education, and lifestyle coaching than usual care. The frequency of medication changes per participant was similar for DMO (mean 0.83, SD 1.49) and usual care (mean 1.00, SD 1.58). At week 4, DMO participants with uncontrolled BP, who were medication adherent (≥80%), appeared to be 4 times more likely than usual care participants to receive an antihypertensive titration. Although this suggests that greater targeting of therapy adjustments may occur in patients who utilize DMO, this finding should be interpreted with caution because the actual number of medication changes that occurred was small and the difference was not statistically significant.

#### Digital Medicine Offering Measurements

The mean ingestion adherence was 86% during the first 4 weeks (combined DMO) and 84% for the entire 12 weeks (12-week DMO) (see Figure 4).

### Exploratory Outcomes

Among statin users, reductions in LDL-C were larger for DMO compared with usual care. These differences were even greater in participants with a baseline LDL-C of 70 mg/dL or higher. In participants with baseline LDL-C of 70 mg/dL or higher (n=54; 4-week DMO: n=6, 12-week DMO: n=28, usual care group: n=20), changes in LDL-C were mean –37.2 (SE 7.9) mg/dL at week 4 and mean –30.1 (SE 8.0) mg/dL at week 12 for DMO and mean –4.0 (4.3) mg/dL at week 4 and mean –10.9 (SE 5.9) mg/dL at week 12 for usual care. The differences in change in LDL-C between DMO and the usual care group were –33.2 (95% CI –50.6 to –15.8) at week 4 and –19.2 (95% CI –36.4 to –2.0) at week 12. These differences were statistically significant (see Table 5 for complete results).

The DMO participants had a nonsignificantly greater increase in PAM score compared with usual care; the changes were mean 7.9 (SE 3.8) for 4-week DMO, mean 7.9 (SE 3.0) for 12-week DMO, and mean 1.7 (SE 3.3) for usual care at week 12 (see Table 6).

**Table 5:** Summary of total cholesterol and low-density lipoprotein cholesterol (LDL-C) outcomes for the combined digital medicine offering (DMO) group only.^a^

Outcome		Usual care	DMO (combined)		
		Value	Value	Difference,^b^ (95% CI)	Adjusted difference,^b^ (95% CI)
**Total cholesterol (mg/dL)^c^**					
	Baseline, mean (SE)	174.4 (13.1)	177.4 (9.5)		
	Week 4, mean change (SE)	–9.2 (7.1)	–34.8 (7.1)	–25.7 (9.7); (–44.6, –6.7)	–23.0 (7.8); (–38.2, –7.8)
	Week 12, mean change (SE)	–21.9 (10.2)	–29.5 (7.4)	–7.9 (11.3); (–30.1, 14.3)	–8.1 (7.9); (–23, 7)
**LDL-C (mg/dL)^c^**					
	Baseline, mean (SE)	99.3 (6.7)	103.9 (10.1)		
	Week 4, mean change (SE)	–3.7 (3.8)	–29.7 (9.4)	–25.6 (9.4); (–44.1, –7.1)	–22.7 (6.1); (–34.6, –10.8)
	Week 12, mean change (SE)	–9.5 (5.6)	–21.3 (10.0)	–11.0 (13); (–37.4, 15.4)	–10.8 (6.3); (–23.1, 1.5)
**Total cholesterol with baseline LDL ≥70 mg/dL (mg/dL)^d^**					
	Baseline, mean (SE)	174.1 (11.6)	185.1 (8.4)		
	Week 4, mean change (SE)	–7.0 (6.9)	–39.8 (7.9)	–32.9 (10.2); (–52.9, –12.9)	–26.5 (9.1); (–44.4, –8.6)
	Week 12, mean change (SE)	–18.9 (9.4)	–37.7 ± 7.5	–18.8 ± 10.6; (–39.6, 2.0)	–13.9 ± 8.8; (–31.2, 3.3)
**LDL-C with baseline LDL ≥70 mg/dL (mg/dL)^d^**					
	Baseline, mean (SE)	104.2 (7.0)	114.0 (7.1)		
	Week 4, mean change (SE)	–4.0 (4.3)	–37.2 (7.9)	–33.2 (8.9); (–50.6, –15.8)	–25.3 (7.0); (–39.1, –11.6)
	Week 12, mean change (SE)	–10.9 (5.9)	–30.1 (8.0)	–19.2 (8.9); (–36.4, –2.0)	–13.4 (7.1); (–27.4, 0.5)

^a^Due to small sample sizes for DMO groups, results are summarized.

^b^Difference from usual care.

^c^Includes participants on any statin therapy in usual care (n=23) and on digital atorvastatin in the DMO group (n=41).

^d^Includes participants on statin therapy in usual care (n=20) and on digital atorvastatin in the DMO group (n=34).

**Table 6:** Summary of Patient Activation Measure (PAM) outcomes.

Outcome	Usual care	DMO (combined)		4-week DMO		12-week DMO	
	Value	Value	Difference,^a^ (95% CI)	Value	Difference,^a^ (95% CI)	Value	Difference,^a^ (95% CI)
Baseline, mean (SE)	70.3 (5.1)	70.6 (2.8)		73.4 (4.7)		68.0 (2.8)	
Week 4, mean change (SE)	–0.9 (1.6)	2.2 (1.9)	3.2 (3.6); (–3.9, 10.2)	2.1 (3.1)	3.0 (4.0); (–5, 11)	2.3 (2.4)	3.3 (3.4); (–3.4, 9.9)
Week 12, mean change (SE)	1.7 (3.3)	7.9 (2.4)	6.2 (4.6); (–2.8, 15.2)	7.9 (3.8)	6.2 (5.3); (–4, 17)	7.9 (3.0)	6.3 (4.7); (–2.9, 15.4)

^a^Difference from usual care.

**Table 7:** Satisfaction survey results (N=75).

Survey question	Answered agree or strongly agree, n (%)
It was easy to use Proteus in my daily routine	68 (91)
It was easy to learn how to use Proteus	69 (92)
Seeing my data showed me how well I’m managing my health	68 (91)
Seeing my data motivated me to improve my health	70 (93)
Proteus helped me have more helpful conversations with my health care professionals	64 (85)
Sharing my data with my health care professionals helped me understand my care plan	68 (91)
Using Proteus improved my experience of health care service for my condition(s)	66 (88)
Proteus helped me to see how I use my medication(s) from day-to-day	66 (88)
Proteus helped me take my medication(s) more regularly	68 (91)
It was easy to use the iPad	67 (89)
It was easy to use the Proteus app	64 (85)
In general, I did not mind wearing the patch	61 (81)
Connecting and applying each new patch was easy for me to do	68 (91)

#### Participant Satisfaction With Digital Medicine Offering

In general, participants agreed the DMO was easy to learn (92%, 69/75) and to incorporate in their daily routine (91%, 68/75), and that using the data was useful to manage (91%, 68/75) and improved their health (93%, 70/75) and that sharing their data with their provider helped them to understand their care plan (91%, 68/75). Most (81%, 61/75) did not mind wearing the patch (see Table 7).

#### Safety

There were 32 of 82 DMO participants (39%) who reported 59 adverse events (AEs), of which 33 were unrelated to the DMO; 8 of 36 usual care participants (22%) reported 17 AEs. There were no serious AEs related to the DMO or the study. There were 14 device-related AEs in 11 participants, most commonly mild skin reactions to the wearable sensor (13 events in 10 participants, 12%). Additionally, 12 participants reported study medication-related AEs (14 events total) with gastrointestinal AEs (9 events in 8 participants; 7 mild, 2 moderate, 1 moderate event led to study withdrawal, 10%) being the most common. Another AE, fatigue (mild intensity), unrelated to the device or study medication, led to study withdrawal.

## Discussion

In this study, participants with uncontrolled HTN and T2DM who used the DMO had significantly greater reductions in SBP within 4 weeks than the usual care group (9 mm Hg greater reduction), which was maintained at 12 weeks with a significantly greater proportion of patients achieving their BP goal. Participants using the DMO also showed lower trends in HbA_1c_ (as much as a 1% greater reduction), and LDL-C (33 mg/dL greater reduction) compared to participants who received usual clinical care. These clinical findings were maintained in analyses adjusting for age, gender, race, and baseline clinical parameters. Use of the DMO was also safe; the frequency of skin reactions is generally lower than published research on adhesive tape and patches [25-28].

These findings are relevant in the care of T2DM patients who have an increased risk of serious cardiovascular and microvascular complications. Each 2 mm Hg reduction in SBP or 1 mm Hg reduction in DBP has been associated with lowering mortality from stroke and ischemic heart disease by 10% and 7%, respectively [29]. A reduction in HbA_1c_ of 0.5% or more is considered clinically significant to reduce the risk of microvascular complications [30,31]. Statin adherence, as evidenced by LDL-C reduction, has also been associated with improved outcomes in diabetes patients [29,32,33]. However, given the short duration of this pilot study, additional longer-term evidence will be necessary to demonstrate that the changes in BP, HbA_1c_, and LDL are durable.

We hypothesize that improved clinical outcomes with the DMO were related in part to improved self-care (medication adherence and patient activation). In the literature, average adherence to chronic medicines is approximately 50%; participants using DMO achieved a mean adherence of 86% during the first 4 weeks. The DMO also had a greater increase in PAM scores; a 1-point increase in PAM score is associated with a 1.8% increased likelihood of decreasing HbA_1c_ to less than 8% [9].

Providers could make more targeted and timely therapy optimization decisions using the objective behavioral data reported by the DMO. We found that at week 4, for participants with uncontrolled BP, investigators in the DMO arm were more likely to make therapy adjustments or give adherence counseling and/or education guided by the DMO data compared to usual care participants.

Other digital health solutions have failed to demonstrate benefit. Bloss et al [34] failed to demonstrate benefits of digital health interventions (telemonitoring devices for BP, blood glucose, or electrocardiogram rhythm) compared to usual care in a large controlled study. In that study, no instructions were given to providers on use of the device data; this has been shown to make blood glucose monitoring less effective in non-insulin-using diabetes patients [30,34]. There were also confounders. For example, many participants with diabetes in the control arm used insulin and likely made insulin-titration decisions based on blood glucose results. Finally, the inclusion criteria were based on cost versus level of disease control and may have enrolled participants not needing additional interventions.

In contrast, a recent study demonstrated benefits of a pharmacist case management intervention to reduce BP using a telemonitoring BP device [35]. Participants in the intervention arm spoke with pharmacists (via the phone) who reviewed BP data, provided coaching, and titrated medications. The intervention group had a statistically greater reduction in SBP at 6 months than usual care (mean difference –10.7, 95% CI –14.3 to –7.3 mm Hg), similar to the reductions in SBP observed in our pilot study on the DMO, except the pharmacist case management study only addressed hypertension, whereas this DMO study evaluated patients with both uncontrolled HTN and T2DM.

There were several limitations to this study. This study had a small sample size and included only 13 sites, which likely contributed to discrepancies noted in participant demographic and baseline characteristics. As mentioned in the Methods, this was a pilot study to measure the effect size of the DMO on SBP. The adjusted analyses suggested that these baseline differences did not affect the outcomes. However, they may have still affected the results for BP at goal. The small sample size may have also contributed to lack of power to detect differences between groups for some of the secondary and exploratory outcomes. In order to demonstrate use of the DMO in the real-world primary care clinic workflow, ambulatory BP monitoring was not used; therefore, BP fluctuations may be potentially related to the context of in-clinic measurement (outside of the participant’s natural context). A comparison of adherence to treatment was not included among the goals of this study. No objective assessment of adherence to treatment was attempted in the usual care arm because there are only indirect methods of measuring adherence as an alternative to DMO and none of these methods have been established to be inherently reliable or accurate. Therefore, we cannot conclude that DMO led to higher levels of medication adherence. However, measuring an improvement in adherence was not an objective of the study.

Despite these limitations, this study demonstrates positive evidence that a digital health offering that measures and promotes medication adherence, patient self-care, and provider engagement can help patients improve their level of BP and diabetes control. The results should be generalizable given the diversity of the study population. Reducing BP, HbA_1c_, and LDL-C in a consistent manner over a longer term through the use of DMO-like approaches may help patients decrease their overall risk for complications. Future real-world evidence can build on these results to further elucidate longer-term outcomes.

## Abbreviations

AE: adverse event
BP: blood pressure
DBP: diastolic blood pressure
DMO: digital medicine offering
FPG: fasting plasma glucose
HbA1c: glycated hemoglobin A1c
HTN: hypertension
ICC: intracluster correlation coefficient
ITT: intention-to-treat
LDL-C: low-density lipoprotein cholesterol
PAM: Patient Activation Measure
T2DM: type 2 diabetes mellitus
